# Analysis of Errors in the Estimation of Impact Positions in Plate-Like Structure through the Triangulation Formula by Piezoelectric Sensors Monitoring

**DOI:** 10.3390/s18103426

**Published:** 2018-10-12

**Authors:** Eugenio Marino-Merlo, Andrea Bulletti, Pietro Giannelli, Marco Calzolai, Lorenzo Capineri

**Affiliations:** Department of Information Engineering, University of Florence, Via S. Marta 3, 50139 Firenze, Italy; eugenio.marinomerlo@unifi.it (E.M.-M.); andrea.bulletti@unifi.it (A.B.); pietro.giannelli@unifi.it (P.G.); marco.calzolai@unifi.it (M.C.)

**Keywords:** structural health monitoring, piezoelectric sensors, plate-like structure, impact positioning

## Abstract

The structural health monitoring (SHM) of critical structures is a complex task that involves the use of different sensors that are also aimed at the identification of the location of the impact point using ultrasonic sensors. For the evaluation of the impact position, reference is often made to the well-known triangulation method. This method requires the estimation of the differential time of arrival (DToA) and the group velocity of the Lamb waves propagating into a plate-like structure: the uncertainty of these two parameters is taken into consideration as main cause of localization error. The work proposes a simple laboratory procedure based on a set-up with a pair of sensors that are symmetrically placed with respect to the impact point, to estimate the uncertainty of the DToA and the propagation velocity estimates. According to a theoretical analysis of the error for the impact position, the experimental uncertainties of DToA and the propagation velocity are used to estimate the overall limit of the SHM system for the impact positioning. Because the error for the DToA estimate depends also on the adopted signal processing, three common methods are selected and compared: the threshold, the correlation method, and a likelihood algorithm. Finally, the analysis of the positioning error using multisensory configuration is reported as useful for the design of the SHM system.

## 1. Introduction

The possibility for identifying damage on a structure by sensory systems allows for the determination of its integrity, thus reducing downtime and maintenance costs. Structural Health Monitoring (SHM) is important because it permits real-time detection of the condition of a structure, particularly of safety-critical components typical of the application of compounds in the space, automotive, and aeronautics sectors [1,2,3,4,5,6,7]. These design goals are also envisaged in multisensory—connected items by the new paradigms introduced by Industry 4.0 [8].

A possible approach used for the SHM consists of two steps: first passive monitoring to detect any mechanical impacts or cracks caused by high mechanical load made by the emission of ultrasonic waves, and then the investigation of the type of damage by the activation of Lamb waves [1,5,9].

The mechanical waves generated by impacts and transmitted into the material can be detected by means of sensors that are positioned on its surface. The ability of a monitoring system to locate the position of the emission source allows it to actively focus the search for damage on a restricted area.

In the literature, different strategies for locating impact positions using Lamb waves are investigated, with particular reference to the extraction of the Differential Time of Arrival (DToA) [10,11,12,13,14,15,16,17,18,19,20,21].

In particular, Tobias in [10] uses a triangulation technique; Ciampa and Meo in [12] evaluate the DToA with an algorithm based on Continuous Wavelet Transform; Shukri Mohd et al. in [13] use a method based on Wavelet Transform Analysis and Modal Location (WTML) with four sensors; Shenxin Yin et al. in [14] work on using eight sensors bonded in a “Z” shaped arrangement. The systems based on neural networks presented by Worden et al. [15] use up to 17 sensors; another empirical approach technique known as DeltaT mapping is studied in [22,23].

The triangulation method introduced by Tobias [10] is widely used in literature. All the triangulation methods are basing on the DToA, and the measured values of the propagation velocity. Moreover, there are other parameters that should be considered for the overall error estimation: the positions of the sensors, the position of the point where the impact occurs and the variation of the velocity with the propagation direction and the homogeneity of the mechanical properties of the plate-like structure. Focusing on the analysis of the DToA and the propagation velocity, it is important to take into account the characteristics of the system implemented, in particular the type of the sensors, the electronic front-end and the data elaboration algorithms. In general, the algorithm used to extract the DToA have parameters adapted to electronic system characteristics such as bandwidth, signal-to-noise ratio, and input signal dynamic. For this reason, is often difficult to compare the impact position accuracy of different methods reported in the literature. The aim of this work is to describe a method for the evaluation of the experimental errors of a targeted set-up, and then with the support of a theoretical analysis, the influence of the different sources of error of the impact position estimation. Without lack of generality, in this work, we present an analysis of errors referring to an acquisition system and an impacts generation tool, while for the signal processing technique we compared three methods: the traditional threshold crossing technique [17,20], the cross-correlation technique [24,25], and a third one implementing the algorithm of "likelihood" previously published by the authors [21]. 

The experimentation was carried out on an aluminum plate with commercial piezoelectric transducers (PZT disc-type Acellent SML-SP-1/4-0) coupled to the plate surface using an ultrasonic shear-wave couplant (Panametrics NPD-053-8002). Only two transducers for the reception of signals have been used and low energy impacts were generated at points on the plate with a custom-built impactor, described in Section 2.

In Section 3, the estimation of the velocity uncertainty was measured at defined frequencies falling in the range of the spectral components generated by the impact, and the results were compared to the theoretical dispersion curves for the A_0_ mode calculated using the Lamb toolbox [26]. In Section 4, the three methods mentioned above to extrapolate the DToA are compared. The analysis of the influence of the DToA and the propagation velocity on the impact point estimation are reported in Section 5, and the results discussed in Section 6.

## 2. Setup for the Generation and Acquisition of Impacts

**Figure 1:**
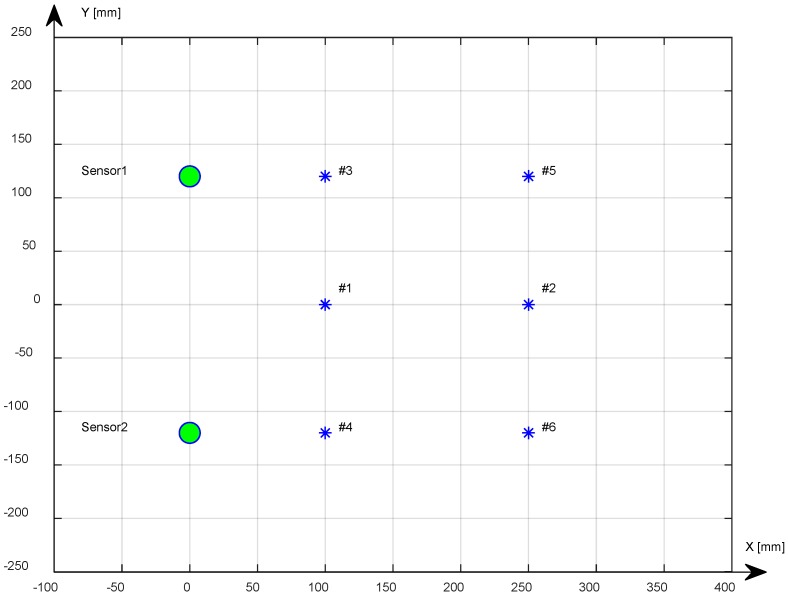
Impact points positions #1, #2, #3, #4, #5, #6 on the aluminum plate. Positions #1, #2 are symmetric respect to Sensor1 and Sensor2.

To conduct the measurements needed to acquire signals coming from the impacts, we used a system composed of an aluminum plate, two receiving sensors (passive mode), and a mechanical system to generate repeated impacts. In Figure 1, the plate schematically shows the points where the two receiving sensors are placed in coordinates (0, 12) cm and (0, −12) cm and the impact points (#1, #2, #3, #4, #5, #6) bonded with ultrasonic shear-wave couplant (Panametrics NPD-053-8002).

The aluminum plate size is 500 mm × 500 mm × 1.4 mm. The piezoceramic-type receiving sensors are manufactured by the PI (Physik Instrumente) model P-876.SP1 DuraAct. The acquisitions of the signals are carried out by directly connecting the sensors to the oscilloscope and acquiring the data with a PC by ethernet link.

The analysis of the errors assumes a set up that capable to reproduce many impact events with known position and energy. Meanwhile it is also necessary to avoid damages to the structure to maintain the same characteristics of the propagating guided wave into the plate-like structure. 

**Figure 2:**
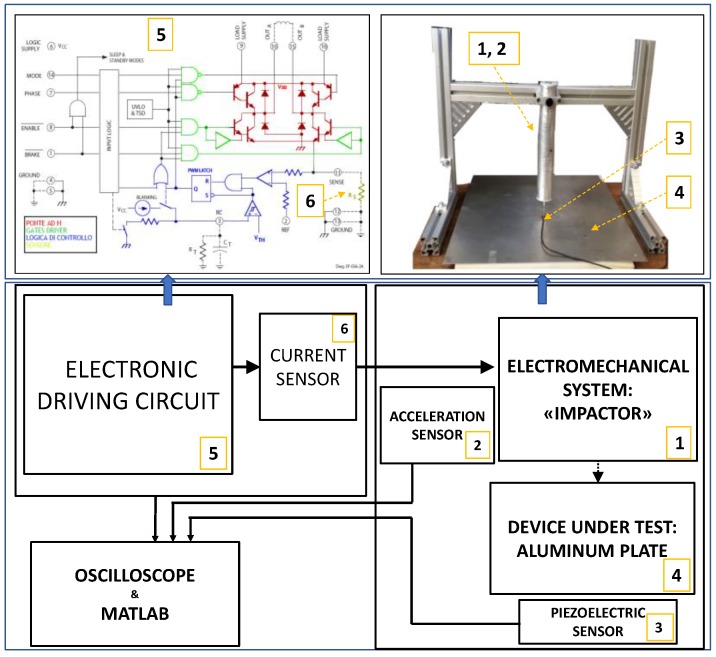
Set-up designed to generate controlled energy impacts: on the left side, the electronics that drives the electro-mechanical device and the instrumentation to acquire and process the data, on the right, the mechanical system that generates impacts and the plate under test.

Therefore, the low energy impactors can be formed by a simple sphere in free fall from a height in the range of tens of centimeters, or by a mechanical pendulum. Besides the complexity of the latter method, the repeatability of the impact position is not ensured, and low energy levels that can be obtained only with very a short acceleration path and low weight spheres. Other methods for the impact generation employs optoacoustic energy conversion with laser sources. This instrument set-up provides very repeatable impacts, but the energy density is very high, and it does not reproduce the actual situation of a spherical body impacting to the structure. Moreover, the cost and safety issues of using laser sources is a disadvantage for adopting this solution. For the aim of this work, we have designed a low-cost mechanical system for generating reproducible impacts with electronically controlled low energy (see Figure 2), to avoid micro damages in the impact zone due to many repetitions. The energy is controlled by using current and acceleration sensors in the control loop. The impact generator comprises of a structure with aluminum struts that supports a voice-coil electromechanical device. The impactor device converts electrical energy into mechanical energy and transfers it to a rigid steel rod with a spherical machined tip with 1mm diameter.

The mechanical system is able to generate the impacts with a controlled force by varying the current flowing in the electromagnetic impactor.

For an accurate positioning of the sensors on the coordinates established in the setup of Figure 1, the center of the sensors has been traced so as to allow their placement in the coordinates (0, 120) (mm) and (0, −120) (mm), achieving a spatial accuracy of less than one millimeter. 

**Figure 3:**
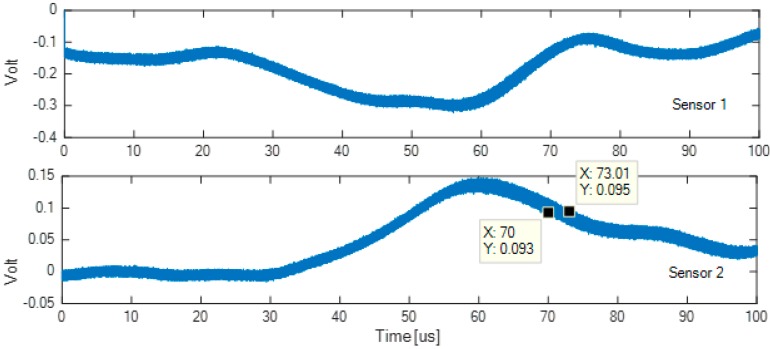
Test on the signal acquisition jitter: acquisition of 100 impacts in the same point of the aluminum test sample, with sensors 1 and 2 shown in Figure 1.

Subsequently, the accuracy of the system for repetitive impacts was evaluated by recording the impacts by carbon paper. The distribution of black dots on the carbon paper sheet has an almost circular area with a diameter of less than 0.5 mm. Finally, Figure 3 shows a 100 µs interval of two signals, acquired and accumulated by sensor 1 and sensor 2 for 100 impacts in the same point, which represents the time and amplitude accuracy of the adopted acquisition system. The oscilloscope used is a Tektronix TDS 3012B 8 bit set with the bandwidth being limited to 20 MHz. The trigger is set to unlimited persistence. During work, the sampling time will be indicated with the symbol Ts.

By observing Figure 3, the two signals acquired by sensor 1 and by the sensor 2 show a time uncertainty of about 3 µs at the point of maximum deviation. It has been estimated that, given the good signal-to-noise ratio at the output of the receiving sensor, for the present measurement, the 50 dB of dynamics provided by the 8 bit resolution oscilloscope was enough for the aim of our analysis.

## 3. Estimation of the Propagation Velocity

**Figure 4:**
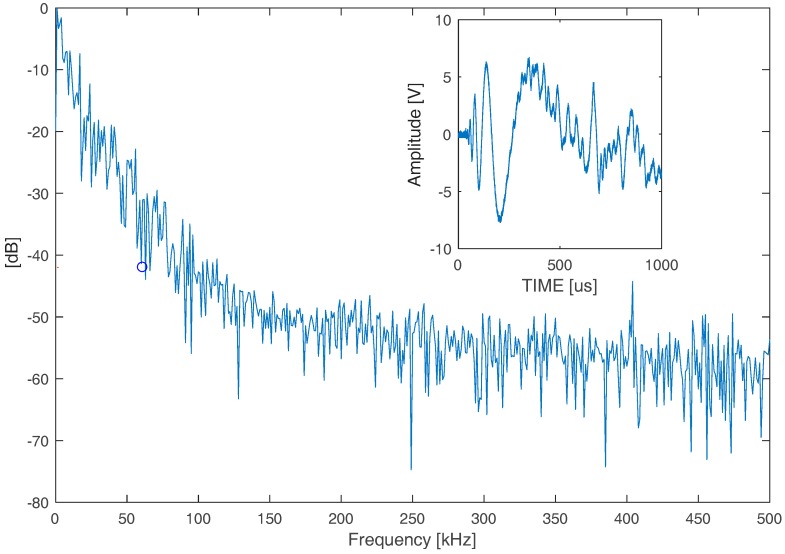
Frequency spectrum of a signal generated by a single impact on the aluminum plate.

Preliminarily, we analyzed a signal that was generated by an impact in the frequency domain to establish the limits of the frequency content in consideration of the signal to noise ratio. To consider our acquisition chain (8 bit resolution), the dynamics of the signals (±10 V), the resolution is 98.4 mV, and we found that the dynamic range is 48.1 dB. According to this system characteristic, we selected a max attenuation of −42 dB for the signal processing and the corresponding maximum frequency is evaluated at around 60 kHz (see Figure 4). This choice is adequate to process the A_0_ mode signal as the main mode that was generated by the impact device. In general, this consideration is important for the SHM system designer, in that it can increase the digital acquisition system resolution to extend the spectral content of the signal and include different propagation modes with lower amplitude. 

The estimation of the velocity was measured at frequencies of 20 kHz, 40 kHz, and 60 kHz using piezoceramic transducers (PZT disc type Acellent SML-SP-1/4-0) coupled to the plate surface using an ultrasonic shear-wave couplant (Panametrics NPD-053-8002), positioned in the test points highlighted in Figure 1, and driven by the Agilent 33220 function generator; data were acquired with the Tek-TDS3012B digital oscilloscope (100 MHz bandwidth).

The velocity dispersion curve of the A_0_ mode Lamb wave in our aluminum plate (1.4 mm thick) was simulated with MatLab using the Lamb Toolbox [26]. 

Experimentally, we estimated the velocity at three discrete frequencies. The measurements were repeated six times and averaged.

**Figure 5:**
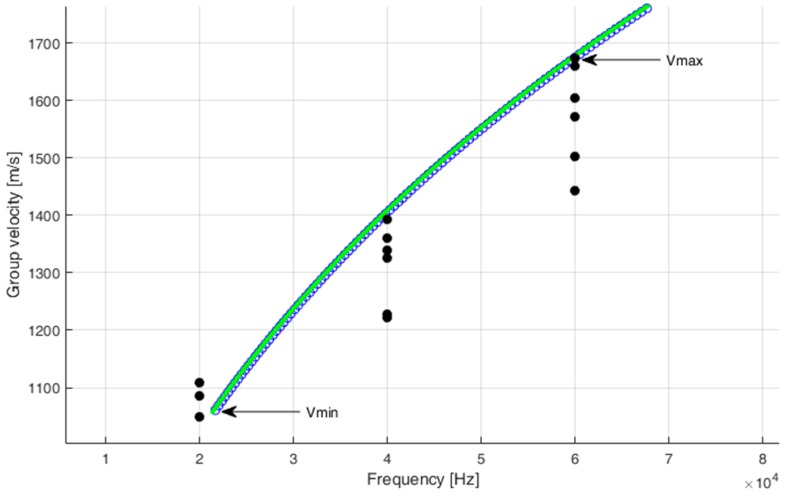
Simulated dispersion curve of the group velocity of the A_0_ mode for 1.4 mm thick aluminum. The measured velocity values for 20 kHz, 40 kHz, and 60 kHz reported with black circles.

The variation respect to average of the estimated velocity, each frequency was 14% for 60 kHz, 12% for 40 kHz, and 17% for 20 kHz. Figure 5 also shows v_max_ and v_min_ representing the minimum and maximum velocities considered in the selected frequency range for an impact signal. These values will be used later in the Section 6 for the influence on the overall error of the position.

## 4. Evaluation Methods of the DToA

The estimation of the DToA, among different techniques [16,21,24,27,28], was performed with the following three methods:

- Threshold,
- Correlation,
- Likelihood algorithm.

Detailed descriptions of the pro and cons of each method are reported in the following by using experimental signal analysis.

### 4.1. The Threshold Method

The threshold criterion is based on the estimation of the time of flight (ToF) corresponding to the exceeding of a predefined value (example six times the noise level) by the signal values.

**Figure 6:**
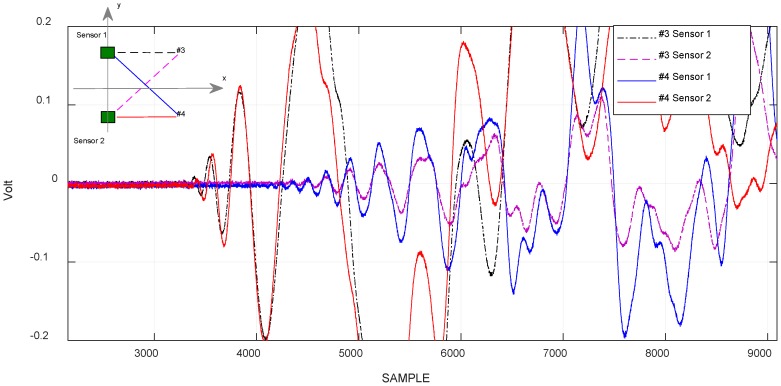
Acquired signals by sensor 1 and sensor 2, generated by an impact, respectively, in positions #3 and #4. Ts = 100 ns.

For the evaluation of the limits of this method, it is necessary to distinguish two cases, according to where the impact test is performed, if it is along the axis of symmetry (positions #1 and #2) of the receiving sensors, or out of symmetry of the same (#3, #4, #5, #6). In order to evaluate the error based solely on measurement data, and not comparing to theoretical values [29], for the first case we calculated the difference between the signal arrival times (dDToA), which was about 10 µs against a null value, while in the second case, the error was evaluated, considering the difference between the DToA calculated between the signals received by the two sensors for impacts generated in the anti-symmetrical positions (#3, #4 or #5, #6). Figure 6 shows the time domain signals for the two impacts in test positions #3 and #4 of Figure 1. The difference between the measured DToA in the respective coordinates was about 13.4 µs.

### 4.2. The Correlation Method

**Figure 7:**
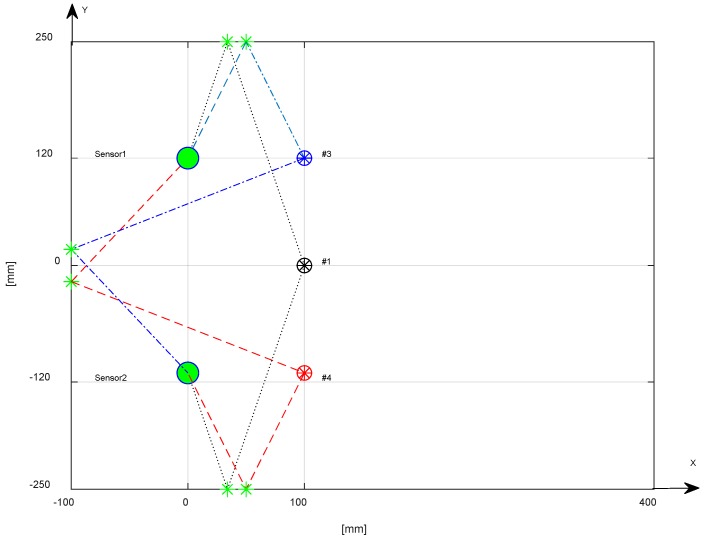
Calculated minimum edge reflection path for impacts #1, #3, #4, with respect to Sensor 1 and Sensor 2.

The correlation criterion highlights the temporal deviation between two signals, and the result indicate the similarities of the whole waveform. In the case of the experimentation of a plate-like structure with limited area, the superposition of the multiple reflection from the edges deteriorates the similarity, especially at later arrival times. In Figure 6, the signals represented are generated by impacts with a voice-coil in some test points. The similarity of the earlier portion of the signals was lost after a certain period of time, because the waves were reflected by the edges overlapping on direct path signal from the impact point to the sensor position. Therefore, it was necessary to apply a time window for the selection of the portion of signal that was not corrupted by multiple reflections. In general, the geometrical calculation of multiple reflections from the edges could be done according to the geometry of the plate-like structure. In our case, for the rectangular aluminum plate, the calculated paths were drawn in Figure 7 relative to the three impacts positions #1, #3, #4.

**Figure 8:**
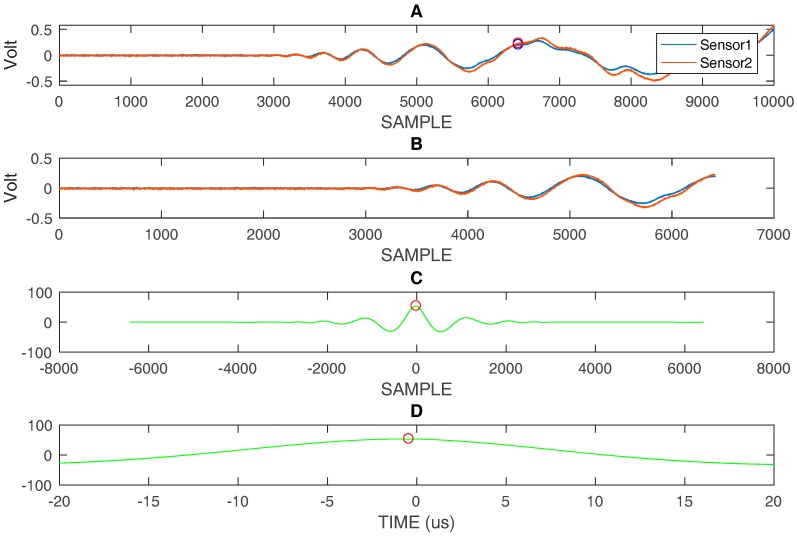
(**A**) impact #1: acquired signal sensor 1 and sensor 2 with marked positions by circles indicating the end of the time window that is used for further processing, (**B**) Selected time window, (**C**) correlation evaluated with the two selected signals shown in (**B**), (**D**) zooming of correlation in (**C**) and representation on time scale: differential time of arrival (DToA) = −0.44 µs. Ts = 40 ns.

**Figure 9:**
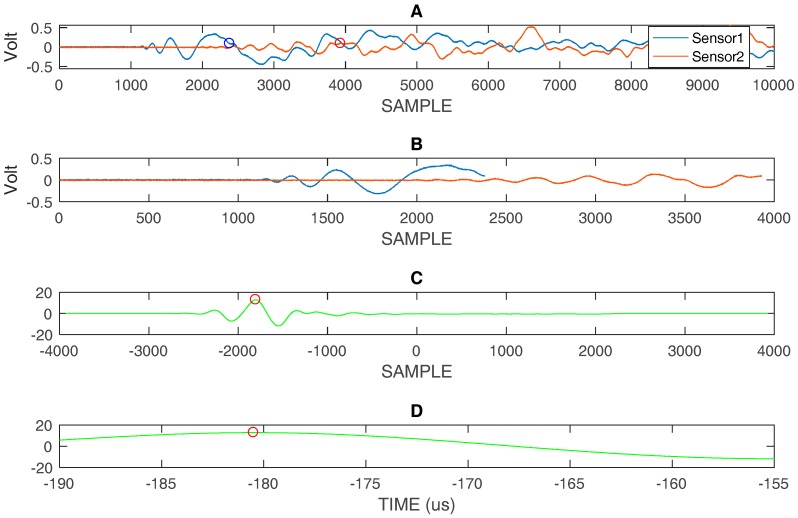
(**A**) impact #3: acquired signals sensor 1 and sensor 2 with marked positions by circles indicating the end of the time window used for further processing, (**B**) selected time window, (**C**) correlation evaluated with the two selected signals shown in (**B**), (**D**) zooming of correlation in (**C**) and representation on time scale: DToA = −180.5 µs. Ts = 40 ns.

**Figure 10:**
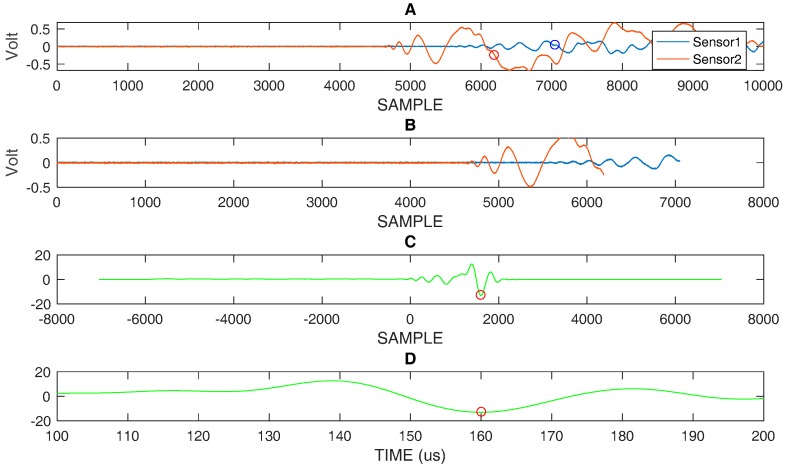
(**A**) impact #4: acquired signals sensor 1 and sensor 2 with marked positions by circles indicating the end of the time window used for further processing, (**B**) Selected time window, (**C**) correlation evaluated with the two selected signals shown in (**B**), (**D**) zooming of correlation in (**C**) and representation on time scale: DToA = 158 µs. Ts = 40 ns.

By converting the distance in time, using the highest velocity of the A_0_ mode (v = 1.63 mm/µs @ 60 kHz) in the range of interest, we can estimate the start of the reflections from edges. In Figure 8A the impact signal in position #1 is shown with a circle indicating the point where the direct signal becomes overlapped with multiple reflection. Figure 8B shows the portion of the signal that is not affected by overlapping for the processing with correlation, and the results, both in graphic and numerical form, are shown in Figure 8C. Similar considerations apply to Figure 9 and Figure 10, which respectively describe the impacts in positions #3 and #4.

The limit obtained for the symmetric position #1 was 0.44 µs, while the difference of the DToA relating to the antisymmetric positions (#3 and #4) was 22.5 µs.

### 4.3. The Method with the “Likelihood” Algorithm

The measurement of the DToA was finally evaluated by applying the algorithm described in our previous work [21]. By estimating the rise time around the zero crossings of the selected portion of the impact signal, a fixed threshold crossing event is considered to be valid only when the corresponding rise time falls within a predetermined range. With this method, we demonstrated the accuracy improvement of the position estimation with respect to the fixed threshold and cross-correlation methods. Moreover, the method is simple and easy to be implement for real-time impact detection on hardware as FPGA (Field Programmable Gate Array).

**Figure 11:**
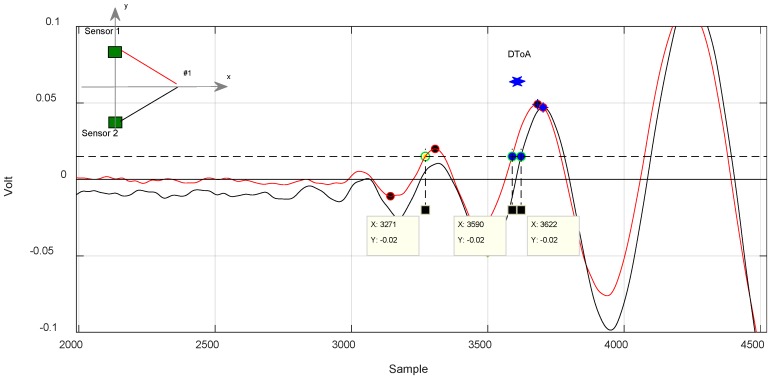
Application of the algorithm to evaluate the DToA in the case of impact along the axis of horizontal symmetry of the sensors. Position #1. Ts = 4 × 10^−8^ s.

**Figure 12:**
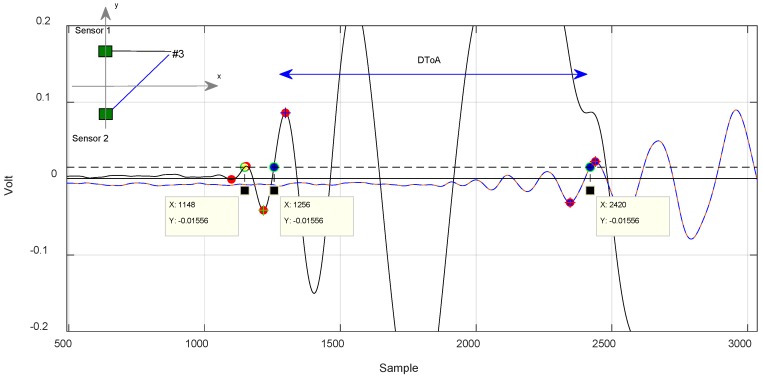
Impact case in position #3, signal received from the sensors sensor 1 and sensor 2. Evaluation method of the DToA with likelihood algorithm. Ts = 1 × 10^−7^ s.

**Figure 13:**
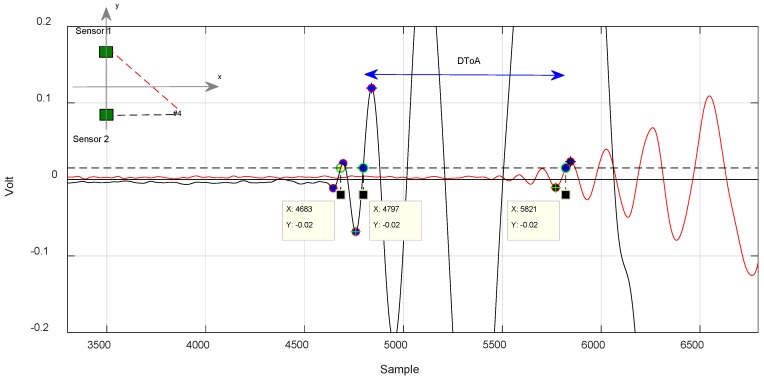
Impact case in position #4, signal received from the sensors sensor 1 and sensor 2. Evaluation method of the DToA with algorithm. Ts = 1 × 10^−7^ s.

For test positions along the axis of symmetry, the method returned 1.28 µs as a limit (Figure 11), while for out-of-symmetry test points, the difference between the two measured DToA results were in the order of 14 µs (Figure 12 and Figure 13).

In Figure 12, we can see how the algorithm works. It selects two similar a very similar portion of the signal corresponding to the trailing edge to calculate the DToA. The yellow dot marks the discarded lobe according to the criterion described previously.

In Figure 12 and Figure 13, it is noted that the algorithm also discards a lobe of the signal in the estimation of the DToA in the case of out-of-axis symmetry impacts.

### 4.4. DToA Results

Table 1 shows the results obtained with the three methods for the extraction of the DToA in the various test positions shown in Figure 1. The reference value is calculated with an estimated average velocity of 1.3 mm/µs.

In positions #3, #4, and in #5, #6 the absolute value of the DToA should theoretically be the same. Differences derived from Table 1 provide an error estimation due to the experimental evaluation of DToA values. In positions #1 and #2, it is possible to estimate the experimental error due to the DToA evaluation, because the DToA values should theoretically be equal to zero.

Table 2 shows the experimental errors that occurred in the evaluation between the DToAs (dDToA).

## 5. Analysis of the DToA and the Propagation Velocity in the Impact Point Estimation

The results obtained in the Section 4 or a couple of sensors can be applied in (1) using the equation introduced by Tobias in [10]:

(1) $$\begin{array}{c} E\left(x_{p},y_{p}\right)=\sum_{i=1}^{N_{T}-1}\sum_{j=i+1}^{N_{T}}|\left(v_{i}t_{i}-v_{j}t_{j}\right)-(\sqrt{{\left(x_{i}-x_{p}\right)}^{2}+{\left(y_{i}-y_{p}\right)}^{2}}-\\ \sqrt{{\left(x_{j}-x_{p}\right)}^{2}+{\left(y_{j}-y_{p}\right)}^{2}})| \end{array}$$

where *N_T_* represents the number of sensors used for the detection of ultrasonic signals, (*x_p_*, *y_p_*) indicates the coordinates of the impact point, and (*x_i_*, *y_i_*), (*x_j_*, *y_j_*) are the coordinates of the sensors considered. *E*(*x_p_*, *y_p_*) represents the value of the *E* function calculated at the considered point.

The times *t_i_* and *t_j_* (with *i*, *j* = [*1*, *N_T_*], *i* ≠ *j*) respectively, indicate the value of the time of arrival of the signal from the instant of the impact (ToA) to the *i*-th and *j*-th sensor, while *v_i_*, *v_j_* are respectively the velocities in the material along the directions that connect the investigated point (*x_p_*, *y_p_*) with the *i*-th and *j*-th sensors respectively.

As it is known, in the Equation (1), there is a minimum value at the point of impact (a zero in ideal case) excluding the areas close to the sensors [10] that is described.

### 5.1. Influence of the Propagation Velocity and the Estimation of the DToA in the Calculation of the Impact Point

Under real conditions, we were not able to estimate *t_i_* and *t_j_*, but only the difference (*t_i_* − *t_j_*) equal to the differential time of arrival.

From Equation (1), we could easily observe that a correct estimate of the impact point necessitated a good estimate of the propagation velocity and the DToA. In case of an aluminum plate, we can consider the uniform velocity in all directions, and therefore we can assume *v_i_* = *v*_j_ = *v*, so that the term (*v_i_ t_i_* − *v_j_ t_j_*) can be simplified:

(2) $$v\left(t_{i}-t_{j}\right)=vDToA_{\left(i,j\right)},\;with\;DToA_{\left(i,j\right)}=\;\left(t_{i}-t_{j}\right),\;i,j=\left[1\ldots N_{T}\right]i\neq j$$

Moreover, in the case of an impact signal with large spectrum components, we need to consider that the propagation velocity is a function of the frequency v=v(f). In Section 3 the estimation of the propagation velocity at defined frequency steps is reported (in our case 20 kHz, 40 kHz and 60 kHz). We can consider a simplified approach in order to understand the influence of the dispersivity, expressing the velocity *v* as an average of the velocity values in the frequency range considered: $v=\bar{v}+dv$, with $\bar{v}=\frac{v_{max}+v_{min}}{2}$ and dv is the deviation velocity with respect to the average velocity ($\bar{v})$. Similarly, for the differential time of arrival (dDToA_ij_) we have an uncertainty of measurement (Table 2) due to the method used. 

Thus Equation (2) can be re-written as:

(3) $$\begin{array}{c} \left(\bar{v}+dv\right)\times \left(DToA_{\left(i,j\right)}+dDToA_{ij}\right)=\bar{v}\times DToA_{\left(i,j\right)}+dv\times DToA_{\left(i,j\right)}+\bar{v}\times dDToA_{ij}+dv\times dDToA_{ij}\\ =\bar{v}*DToA_{\left(i,j\right)}+e_{1ij}+e_{2ij}+e_{3ij} \end{array}$$

$$\begin{array}{c} e_{1ij}=dv\times DToA_{\left(i,j\right)}\\ e_{2ij}=\bar{v}\times dDToA_{\left(i,j\right)}\\ e_{3ij}=dv\times dDToA_{\left(i,j\right)} \end{array}$$

In Equation (3), it is clear that the calculation $\bar{v}*DToA$ adds three distinct error terms: $e_{1ij}$, $e_{2ij}$, and $e_{3ij}$. They depend on the dispersion of the velocity values, and on the uncertainty on the measurement of the differential time of arrival.

The influence of the three errors $e_{1ij}$, $e_{2ij}$, $e_{3ij}$ on the final accuracy for the identification of the point of impact will be thoroughly considered in the discussions after having analyzed the single parameters included. The influence of the dispersion of the velocity values in Section 5.2 and the experimental estimate of the DToAs will be evaluated with a simulation to judge the contribution of the errors.

### 5.2. Influence of the Velocity Variation and DToA at the Point of Impact

To analyze the factors that affect the accuracy of the impact positioning, with the triangulation formula, we consider only one pair of sensors (*N_T_* = 2) and set *E* equal to 0 in (1):

(4) $$\left(\bar{v}+dv\right)*DToA_{\left(1,2\right)}-\left(\sqrt{{\left(x_{1}-x_{p}\right)}^{2}+{\left(y_{1}-y_{p}\right)}^{2}}-\sqrt{{\left(x_{2}-x_{p}\right)}^{2}+{\left(y_{2}-y_{p}\right)}^{2}}\right)=0$$

Equation (4) represents the equation of a hyperbola, with the foci coinciding with the two sensors (*x*_1_, *y*_1_), (*x*_2_, *y*_2_). By a simple MatLab script, the nature of the influence of the velocity variation on the point of impact according to the area of the considered plane was assessed. An exact DToA was considered for a velocity of 1.3 mm/µs, taken in the interval [*v_min_*, *v_max_*], then fixing the DToA value and varying the velocity in the range ±0.3 mm/µs.

**Figure 14:**
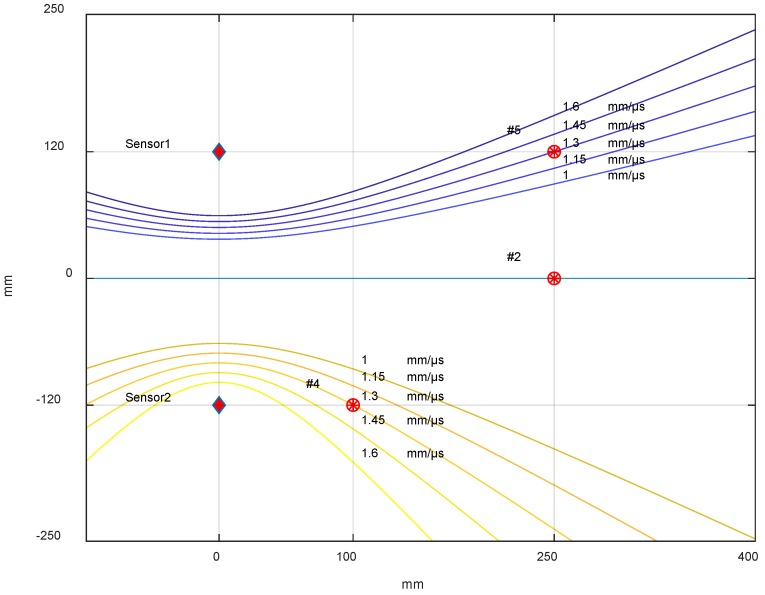
Graphical results derived from Equation (4) with an average velocity of 1.3 mm/µs and its variation in the range of ±0.3 mm/µs. At symmetric impact point #2, the curves are overlapped for all velocities, showing independence from velocity values.

Figure 14 shows that at a greater distance from the pair of sensors, the estimate of the impact point depended on the velocity used in the triangulation formula. Obviously, in the case where the impact point was in a symmetrical position with respect to the two receiving sensors, the variation in velocity was not affected, as the differential time between the two sensors was zero. In the latter configuration, we were able to evaluate the minimum error committed; below this value, further experimental measurements were not possible. The result in position #3, #6 are not shown because they are specular to position #4, #5 and the result in position #1 is the same as in position #2.

**Figure 15:**
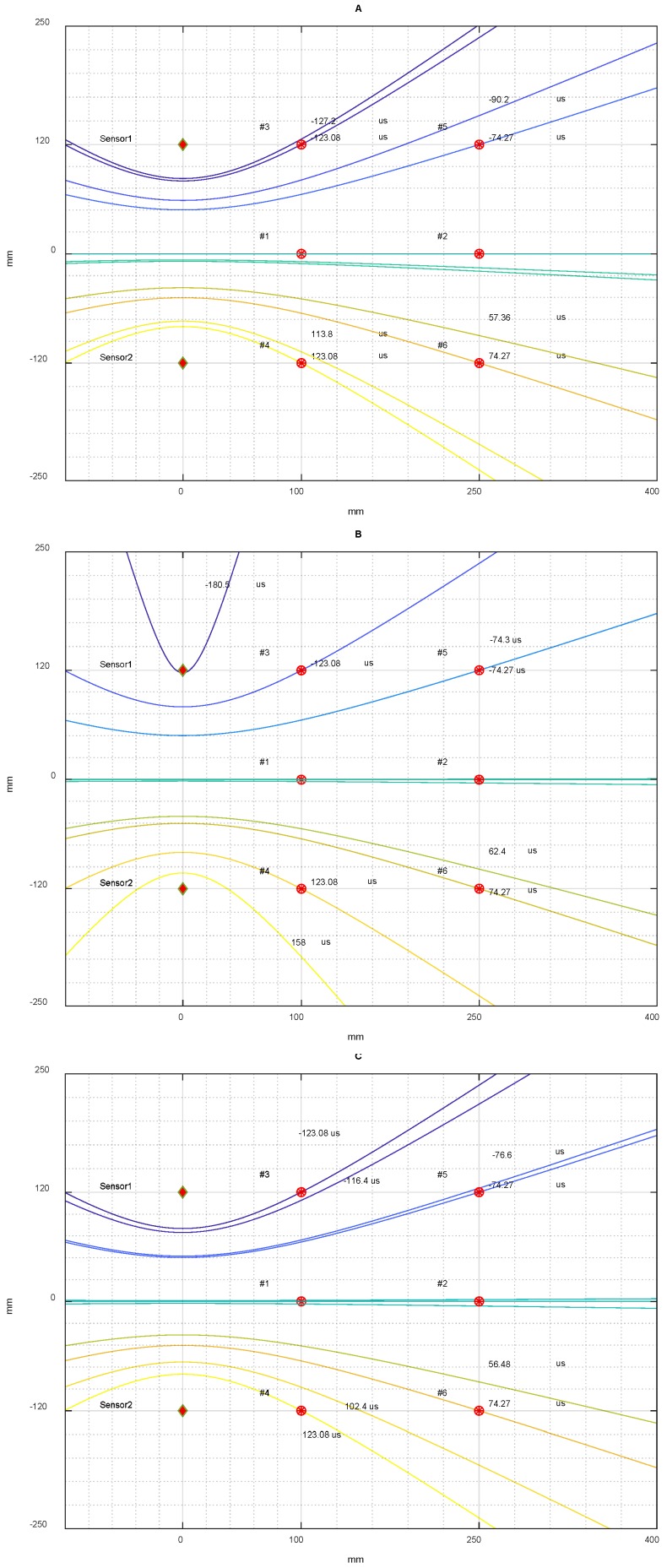
Graph of (4) drawn with a base velocity of 1.3 mm/µs and two DToAs for each point (#1, #2, #3, #4, #5, #6). The first DToA is theoretically estimated (curve intersect in the red point) and the second DToA is calculated with: (**A**) the threshold method, (**B**) the correlation method, (**C**) the likelihood method.

Figure 15, shows the influence in Equation (4) of an error on the DToA (see Table 1) with respect to the exact DToA using the mean velocity of 1.3 mm/µs.

## 6. Discussion

By the analysis of Equation (3), it can be noted that the error $e_{1ij}$ takes into account the variation of the velocity with the value of the DToA, and it depends on the position of the impact: if the impact occurs at the same distance from the two reference sensors, the DToA is theoretically null, and therefore the contribution of velocity variation is negligible. In our experiments, we obtained the maximum value of the DToA in positions #3 and #4: in these positions, for variations of velocity of ±0.3 mm/µs, we have maximum error of about 37 mm. Figure 14 shows the influence of this error in the estimation of the propagation velocity.

Otherwise, the $e_{2ij}$ error depends on the method that is used to estimate the DToA and on the position of the impact. In our experiments, the variation of this error is in the range varying from 1 mm to 43 mm. This error is shown in Figure 15 for each point considered.

Finally, the analysis shows that the error $e_{3ij}$ is the combination of the errors $e_{1ij}$ and $e_{2ij}$. In our experiments, this error is always evaluated to be less than 10 mm.

## 7. Conclusions

In this research, we have investigated the limits of accuracy on the detection of the position of impacts with the use of an aluminum plate, using the method of triangulation with Lamb waves, and we then analyzed the results obtained with impacts performed along the axes of symmetry and on asymmetric positions with respect to a pair of piezoelectric sensors.

In order to implement the triangulation method, we evaluated the DToA with three methods, and estimated the propagation velocity of the Lamb waves propagating in the plate-like structure.

After having designed an electromechanical system for the generation of controlled energy impacts with high accuracy of position (better that 0.5 mm), we have identified three causes of errors in the triangulation method that influence the estimate of the impact point positioning.

To obtain an estimate of accuracy, we have minimized the potential factors that could alter the signals generated by impacts, and we used a measurement setup identical for all measurements (velocity and extraction of the DToA). To evaluate the uncertainty of DToA, we have theoretically analyzed the errors that are related to the choice of sensor positions with respect to the impact positions; the results of the investigation is also useful for defining the characteristics of the experimental set up. The outcome is a distribution of the impact points with symmetric geometry, allows to for reliable evaluation of the variability of the DToA.

The choice of an isotropic and homogeneous material such as aluminum has allowed for the reduction of some experimental uncertainties that are usually encountered during the investigation of the point of impact in the materials as the dependence of the propagation velocity from the direction. The error due to the assumption of a single value for velocity has been evaluated, and it is shown that it can be minimized by selecting the portion of signal that possesses similar frequency characteristics by an adequate algorithm. Other methods such as dispersion compensation can also be applied and compared to the standard one for the evaluation of the DToA.

The DToA estimation was performed with three methods: the first two—with a fixed threshold and with cross-correlation—have been selected as the ones most frequently used in the literature, and a third called the likelihood algorithm, developed by the authors. In this way, we can quantitatively evaluate the improvements obtained by developing more sophisticated processing algorithms compared to a set of reference methods.

In our experimentation, the uncertainty on the propagation velocity due to dispersivity in the frequency range up to 60 kHz determined by the characteristic of our system, is equal to 300 m/s with an average value of 1300 m/s.

In an experimental system of impacts positioning on a plate-like structure as reported in this study, we expect an error in the estimation of the impact point position determined by three components $e_{1ij}$, $e_{2ij}$, $e_{3ij}$. These errors are due to the variation of the velocity ($e_{1ij})$, to the method that is used to estimate the DToA ($e_{2ij})$, and the last error is due to the combination of the variation of the velocity and to the method that is used to estimate the DToA $\left(e_{3ij}\right)$. The error ($e_{1ij}$) varies from 0 mm to 37 mm, $(e_{2ij}$) varies from 1 mm to 43 mm, and $\left(e_{3ij}\right)$ from 0 mm to 10 mm. The approach based on the estimation of the three errors $e_{1ij}$, $e_{2ij}$, $e_{3ij}$ can also be applied to different acquisition systems based on different front-end electronics (e.g., resolution of acquisition, bandwidth) and elaboration techniques.

Finally, the proposed method can be extended also to a multi-sensor SHM system for each pair of sensors.

## Figures and Tables

**Table 1:** Results of DToA obtained by the three methods.

Test Points	DToA IMPACTS		
	Threshold	Correlation	Likelihood Algorithm
	(µs)		
Symmetric distance	Reference value 0		
#1	12.7	−0.44	−1.28
#2	10.24	2.4	3.4
Asymmetric distance	123.08 µs @ v = 1.3 mm/µs		
#3	−127.2	−180.5	−116.4
#4	113.8	158	102.4
	74.27 µs @ v = 1.3 mm/µs		
#5	−90.2	−74.3	−76.6
#6	57.36	62.4	56.48

**Table 2:** Evaluation of the differences between DToAs (dDToA).

Test Points	dDToA		
	Threshold	Correlation	Likelihood Algorithm
	(µs)		
#1	12.70	0.44	1.28
#2	10.24	2.40	3.40
#3–#4	13.40	22.50	14
#5–#6	32.84	11.90	20.12
